# Identification of Thermophilic Aerobic Sporeformers in Bedding Material of Compost-Bedded Dairy Cows Using Microbial and Molecular Methods

**DOI:** 10.3390/ani11102890

**Published:** 2021-10-04

**Authors:** Isabella J. Giambra, Yeasmin Jahan, Tong Yin, Petra Engel, Christina Weimann, Kerstin Brügemann, Sven König

**Affiliations:** Institute of Animal Breeding and Genetics, Justus-Liebig-University of Gießen, Ludwigstr. 21b, 35390 Giessen, Germany; yeasminr15@gmail.com (Y.J.); tong.yin@agrar.uni-giessen.de (T.Y.); Petra.Engel@agrar.uni-giessen.de (P.E.); Christina.Weimann@agrar.uni-giessen.de (C.W.); kerstin.bruegemann@agrar.uni-giessen.de (K.B.); sven.koenig@agrar.uni-giessen.de (S.K.)

**Keywords:** compost-bedded pack barns, thermophilic aerobic sporeformers, 16S rRNA-gene sequence, TAS amount, TAS species, moisture content

## Abstract

**Simple Summary:**

Compost-bedded pack barns (CBP) reflect a novel dairy cattle housing system with favourable effects on animal health and animal behavior but can promote the growth of thermophilic aerobic sporeformers (TAS) in the composting lying surface. In our study, we determined a medium–high mean TAS concentration across all bedding samples of four different CBP groups. Six different TAS species were identified based on their 16S rRNA-gene sequence, with *Bacillus licheniformis* being the predominant species. Season, the moisture content of the bedding material and the relative humidity above the bedding material had significant influences on the amount of TAS in the bedding material of the CBP. In addition, the moisture content and the relative humidity above the bedding material significantly influenced the concentration of TAS species each. Other characteristics such as the bedding temperature, the bedded area/cow and the usage time of the bedding material had slight effects on the TAS species occurrence. Due to the negative effect of TAS on milk product quality, considering all identified farm characteristics to optimise TAS contents will contribute to sustainable CBP farming.

**Abstract:**

Compost-bedded pack barns (CBP) are of increasing interest in dairy farming due to their positive effect on animal welfare. The temperature and the moisture content of the bedding material characterising the composting process can promote the growth of thermophilic aerobic sporeformers (TAS). Therefore, the aim of this study was to determine CBP bedding material characteristics, such as moisture content and temperature, and to determine TAS species. The dilution, the heat inactivation of all non-TAS species and the incubation of 13 bedding samples from four CBP groups resulted in a mean TAS amount over all samples of 4.11 log10 cfu/g bedding material. Based on the subsequent sequencing of parts of the 16S rRNA-gene of 99 TAS colonies, the TAS species *Aneurinibacillus* *thermoaerophilus*, *Bacillus* *licheniformis*, *Geobacillus* *thermodenitrificans*, *Laceyella* *sacchari*, *Thermoactinomyces* *vulgaris* and *Ureibacillus* *thermosphaericus* were identified. The moisture content of the bedding material, the relative humidity above the bedding material and the sampling season significantly affected the amount of TAS. The moisture content or relative humidity above the bedding material significantly influenced the concentration of *Ureibacillus thermophaericus* or *Laceyella* *sacchari*. Consequently, an optimal CBP management including a dry lying surface and an optimal composting process will contribute to a moderate microbial, especially TAS amount, and TAS species distribution.

## 1. Introduction

During the past decade, compost-bedded pack barns (CBP) received increasing attention as a housing system for dairy cows due to its potential to improve animal welfare [1,2]. A CBP consists of a large, open resting area, where cows have access to a minimum of 7.40 m^2^/cow and an ideal of 9.00–10.00 m^2^/cow open bedded pack area, usually bedded with sawdust, dry fine wood shavings, spelt husks or miscanthus grass [1,3,4]. Open resting areas without boxes and partitions as installed in classical cubicle and tie-stall housings [5] are generally separated from a concrete feed alley by a retaining wall. The term freewalk housing has been used to describe this system [4,6,7], because cows can walk freely within a barn.

Especially improved comfort during resting, better foot and leg health, more natural animal behavior, and improved manure quality have been described in comparison to in tie-stall barns or cubicle housings [2,7]. According to Blanco-Prenedo et al. [7], incidences for disease and welfare indicator traits from freewalk housings are smaller than in cubicle systems. For example, freewalk cows showed fewer hairless patches in most body areas and fewer lesions in the lower hind legs than cows from the control groups. Regarding lying behaviour, cows in freewalk barns adopted comfortable lying positions more frequently, took less time to lie down, had less difficulties to rise up and had fewer collisions with the barn facilities and herd mates than cows kept in cubicle systems [7]. As lameness is largely recognized as one of the most important problems in modern dairy farms [7,8] and comfort in resting areas improves lying time with further impact on milk production [9], economic importance is clearly given. Specifically, housings in CBP positively influence milk yield [10,11] as well as milk composition traits and milk product quality [12]. Furthermore, it is expected that housings in bedded pack barns will contribute to increased longevity [13].

The bedded pack is a mixture of organic bedding materials and cattle excreta, which is cultivated several times per day. Bedding costs are generally high but may be compensated through reduced lameness, increased milk production and favourable impact on longevity [10]. In addition, the faeces and the urine of cows absorbed or mixed in the pack can be used as solid manures. Typically, in CBP, the pack is renovated in intervals of 6 to 12 months [1,10,14], implying that the bedded pack can provide manure storage for a quite long period.

Cultivation aerates the accumulated pack and mixes the manure and urine of the surface into the pack to provide a fresh bedding for cows to lie down [3]. Aeration enhances the activity of microorganisms in the pack. Accordingly, an aerobic heat initiating the composting process is promoted [2,3,11].

The temperature and moisture content of the bedding [15] as well as microbial diversity [16] are important parameters for composting efficiency. Ideally, in CBP, the internal pack temperature at depths of 15 to 31 cm ranges from 43.30 to 65.00 °C [1,17]. Compost temperatures above 55 °C promote sanitisation, but temperatures between 45 and 55 °C maximise material degradation [18]. For low temperatures between 35 and 40 °C, the diverse microbial population hampers the composting process [18]. However, the optimal temperature range is only partly fulfilled [10,11]. Especially in winter months, frequent aeration may result in a heat loss from the pack, thus disturbing the composting process [14]. However, the difference between the pack and air temperatures as measured on some farms in winter indicates that the pack is biologically active [2].

Increasing temperature can also promote the evaporation of excreta water [2]. Ideally, the combination of manure and substrate should not exceed a moisture content of 70% [11], although a range of 40% to 60% is preferred [3,17,19]. Furthermore, the supplement with dry bedding materials or mechanical ventilation can be used in CBP to promote pack drying. Consequently, Leso et al. [20] recommended an optimal ventilation management, which allows a high drying rate of bedding, even at lower bedding temperatures when the biological activity of microbial population and heat production is reduced.

Additionally, increasing internal compost temperatures are associated with decreasing levels for *Staphylococci*, *Streptococci* and *Bacilli* species in the pack area [5], explaining a decreasing mastitis infection rate [10]. However, as one of the objectives of CBP management is to maintain active composting by providing adequate conditions for bacteria growth in the pack, total bedding bacterial counts in CBP are quite high (ranging from 7.00 to 8.90 log10 cfu/g; [2]). High pack temperatures in CBP are advantageous for the development of thermophilic aerobic sporeformers (TAS; [13]), a group of microbes that are capable of growing at high temperatures. The optimum growth temperatures of TAS are usually between 50 and 65 °C but vary between species and strains [21]. Since bedding materials in CBP provides microenvironments to control the spore viability of TAS, the influence on the number of TAS in cow milk is hypothesised [22]. Therefore, detailed analyses of the bacterial contamination of CBP, especially of TAS, are of great interest.

Taxonomic studies have provided evidence that TAS are very diverse [23]. Studies of the 16S rRNA-gene sequences, for example, have revealed a high degree of heterogeneity and led to the reclassification of thermophilic members of the genus *Bacillus* such as *Geobacillus*, *Ureibacillus* and *Aneurinibacillus* [24,25]. Several studies addressed the contamination of bedding materials in barns with TAS. Driehuis et al. [26] showed that *Bacillus thermoamylovorans* was the most abundant TAS species in the compost samples in dairy cow barns after a heat treatment of 30 min at 100 °C, followed by *Bacillus licheniformis*, *Geobacillus thermodenitrificans* and *Ureibacillus thermosphaericus*. Accordingly, Wang et al. [27] identified *Bacillus licheniformis*, *Geobacillus thermodenitrificans* and *Ureibacillus thermophaericus* in poultry and cattle manure composts. Furthermore, the TAS species *Laceyella sacchari* and *Thermoactinomyces vulgaris*, leading to respiratory diseases in farm animals, are frequently isolated from outdoor environments such as soil and grassy pastures as well as from mouldy hay, grain and horse manure [28]. *Bacillus licheniformis* is wildly distributed in the cattle environment and is detected, e.g., in fodders and in faeces. Spores in silage are unaffected during the gastrointestinal passage and are excreted in faeces [29,30], thus contaminating the CBP bedding material. In causality, a contaminated bedded pack can contaminate cow teats, because lactating cows spend 12 to 14 h/d lying down in direct contact with bedding [31], and can be transferred into milk [32]. Hence, *Bacillus licheniformis* is also detected in raw milk and in pasteurised samples [21,29,32,33,34,35]. As TAS can survive after heat treatment during the pasteurisation process, they are one of the most common spoilage-causing microflorae in milk and dairy products. Furthermore, they produce enzymes and acids that may lead to off-flavours in the final milk product [36,37].

Sequence analyses of the highly conserved ribosomal RNA genes (rRNA), in particular of the 16S rRNA-gene, are used to identify bacterial species in clinical practice and scientific studies [38,39]. Consequently, 16S rRNA-gene sequence analysis is established as a key technique to identify microorganisms [23,40]. Bacterial 16S rRNA-genes contain nine hypervariable regions (V1–V9), which differ in length, position and taxonomic discrimination [39,41]. In bacteria, hypervariable regions are flanked by conserved stretches, enabling the polymerase chain reaction (PCR)-amplification of target sequences with universal primers. Chakravorthy et al. [39] indicated that the hypervariable regions V2, V3 and V6 contain the maximum nucleotide heterogeneity, implying the maximum discriminatory power for the bacterial species.

The aim of the present study was to measure the CBP bedding parameters moisture content, the bedding temperature, the temperatures and the relative humidities at the heights of 0.10 and 1.30 m above the bedding material, and the ambient temperature of one German dairy farm in different sampling seasons for different animal groups with different lactation status and a variable bedded area per cow.

Thus, the main objectives of the study were to estimate the effects of the CBP bedding parameters on the TAS amount and the TAS species distribution under practical conditions.

## 2. Materials and Methods

### 2.1. Sample Collection

The bedding material of one dairy CBP farm located in Hesse, Germany, was collected on five different dates during December 2017 and February 2019. During this time, 726 to 790 dairy cows of the breed German Holstein were kept on the entire farm. Four cow groups from this farm reflected CBP, whereas each CBP group was housed in its own pen. Dry cows (CBP4-dry), fresh-lactating cows (CBP1-lactating (lact.)) and two high-performance groups (CBP2-lact. and CBP3-lact.) were kept in the CBP. First-lactation and late-lactating cows were kept in a cubicle barn on the same farm. Bedded areas per cow in CBPs were on average from 7.80 to 21.96 m^2^/cow, depending on the group size at the corresponding sampling date, whereas dry cows had a more space per cow in general. Depending on the number of seasonal calvings, different numbers of cows were kept in the different groups (CBP1-lact.: 105–119 animals; CBP2-lact.: 98–150 animals; CBP3-lact.: 110–145 animals; CBP4-dry: 42–84 animals), explaining the differences in the bedded area/cow (m^2^) at the different sampling dates (Table 1). Cows receiving antibiotics in case of mastitis or further diseases were kept in a separate pen and were not included in the study.

The roughage components (silage) were the same over all groups, only differing across seasons; concentrate feeding was milk-performance-related.

The average depth of the bedding material in the different groups of CBP was 50 cm including a mixture of cereals husks, wood chips, miscanthus mulch and sawdust. Depending on the availability, also shredded brushwoods and broken roots were bedded. The bedding material was cultivated three times per day during milking with a rotary tiller (in the morning and evening) or a field cultivator (at midday) at a depth of 20 cm to incorporate excreta and to ventilate the bedding material. In this process, oxygen entered the bedding material and promoted the aerobic microbial conversion. In addition to fans, a patented installed suction device under the bedding material extracted warm and humid air at the bottom.

The corresponding sampling took place at midday, shortly before the next mechanical handling. Aliquots of the bedding material were collected at a depth of 20 cm by scraping compost with a little shovel at nine different sampling spots, evenly distributed throughout the entire stable group (minimum 1 m from the outer walls) according to Leso et al. [42]. For dry cows, three different sampling sites were chosen following the same scheme. These aliquots were thoroughly mixed to create a composite sample representative for the entire CBP at this sampling date, considering a minimum of 500 g taken in a plastic jar. The samples were transported at ambient temperature to the laboratory of the Institute of Animal Breeding and Genetics, JLU Gießen, Germany. Moisture content was determined, and the samples were stored in the laboratory at −20 °C until further analysis.

In total, 13 different samples of the bedding material from the four different CBP groups on this farm were collected at five different sampling dates in four seasons (for details see Table 1). Seasons were defined as follows: 20 March, the first day of spring; 21 June, the first day of summer; 22 September, the first day of autumn; and 21 December, the first day of winter.

During the first sampling in winter 2017, the groups CBP2-lact. and CBP3-lact. were not housed. Therefore, only CBP1-lact. and CBP4-dry were considered in the sampling scheme, which explained the differences in intervals between the complete renewal of bedding and the sampling day in spring 2018 (Table 1). In further samplings, we aimed at a standardised sampling scheme regarding environmental and farm management influences. However, at some sampling dates, the farm management substantially differed, e.g., due to cultivation processes. In such cases, we omitted the collection of samples for TAS analyses.

In order to obtain an overview of the farm-specific contamination with TAS, a sample of the bedding material from the lying boxes in the cubicles was additionally collected in spring 2018. Furthermore, one sample was taken from the unused bedding material from the bedding store in winter 2017.

Pack depth temperatures (−0.20 m) as well as the temperatures and relative humidities at the heights of 0.10 and 1.30 m above the bedding were measured for each of the 9 evenly distributed sampling locations using a temperature sensor thermometer testo 435 (testo SE & Co. KGaA, Lenzkirch, Germany).

### 2.2. Sample Preparation

According to Driehuis et al. [43], 20 g of each compost sample were added to 180 mL distilled water and were mixed thoroughly for 1 h. A volume of 5 mL of this sample extract was heated at 100 °C for 30 min in a hot water bath chamber, leading to the death of all non-TAS species. Afterwards, the sample extract was immediately put on ice. Then, 4.50 mL of 6.44 mM Chloride-Peptone Solution (PanReac AppliChem) were used to dissolve 500 µL of the initial sample extract. Afterwards, the dissolved extract was used for a four-fold serial dilution [43]. In the next step, 1 mL of each dilution was plated on a Dextrose Tryptone Agar (DTA; Oxoid LTD, Basingstoke, UK) plate and incubated at 55 °C for 48 h. Negative control tests were performed each time by co-incubating uninoculated agar plates. Colonies detectable on a DTA medium after the heat treatment described above were defined as colonies derived from reactivated TAS spores according to Driehuis et al. [26].

### 2.3. Counting the Inoculated Agar Plates

Following incubation at 55 °C for 48 h, grown TAS colonies were counted, and the number of colony-forming units per gram of bedding material (cfu/g) was calculated with the following equation:

cfu/g = [∑c/(1 × n^1^ + 0.1 × n^2^)] × d, (1)

where ∑c is the sum of all counted colonies; n^1^ is the number of plates of the first dilution stage. In this dilution stage, the grown colonies were countable for the first time; n^2^ is the number of plates of the second dilution stage. This was the following dilution stage after the specified first dilution stage; d is the dilution level of the first counted plate. 

The data were finally logarithmised (log10) to create an approximate normal distribution.

However, it was not possible to determine individual bacterial species on the basis of the grown colonies. Consequently, we sequenced parts of the 16S rRNA-gene of the grown TAS colonies.

### 2.4. Amplification of Parts of the 16S rRNA-Gene Sequence of Grown TAS by PCR

After incubation at 55 °C for 48 h, in total 99 grown colonies (1 to 22 colonies per bedding sample; Table 1) were picked for colony PCR without the step of DNA extraction [44]. Therefore, a single bacterial colony was picked with a pipet tip and rinsed in 40 µL of distilled water prepared in separate tubes. Afterwards, the picked and rinsed colonies were incubated at 95 °C for 5 min, cooled down and centrifuged at 3000 rpm for 1 min. The supernatant was collected and used as a template for adjacent PCR. This was also performed for the bedding sample of the cubicles. Here, 11 colonies were picked.

According to Chakravorty et al. [39], who proposed the hypervariable regions V2, V3 and V6 of the 16S rRNA-gene as most suitable for bacteria species identification, we decided to amplify these variable regions by two separate PCR using the primers specified in Table 2. PCR included 10 pmol of each primer (Microsynth AG, Balgach, Switzerland), PCR-buffer (Promega, Mannheim, Germany), 2 mM MgCl2 (Promega), 0.25 mM dNTPs (Thermo Fisher, Germany) and 1 U of Taq-polymerase (Promega, Germany). PCR amplification was carried out for both PCR as follows: after the initial denaturation of 10 min at 95 °C, the temperature cycling was in the following way: 30 cycles at 95 °C for 20 s, at 52 °C for 30 s, at 72 °C for 30 s and a final extension step at 72 °C for 5 min.

The resulting PCR products were quality-controlled by agarose gel electrophoresis and using a Nanodrop 1000 Spectrophotometer (Peqlab, Erlangen, Germany) and afterwards, purified using an MSBSpin PCRapace kit (Invitek Molecular GmbH, Berlin, Germany) according to manufacturer’s instructions. The purified colony PCR products were used for subsequent sequencing reactions using one of the PCR primers. The sequencing of the 16S rRNA-gene regions V1–V3 and V6 were made using an Applied Biosystems 3130 Genetic Analyzer (Applied Biosystems, Thermo Fisher Scientific Inc., Waltham, MA, USA).

Afterwards, the resulting sequences were analysed using ChromasPro 1.32 (Technelysium Pty Ltd., Queensland, Australia). Searches for the sequence similarity of known bacterial species were performed using BLASTn [45] accessed via the National Center for Biotechnology Information (NCBI) website (https://blast.ncbi.nlm.nih.gov/Blast.cgi (accessed on 20 April 2021)). Only alignments with a minimum sequence similarity of 99.65% were included for further studies.

### 2.5. Statistical Analyses

Association analyses were performed by applying the linear model function (lm) in R version 4.0.2 [46]. In this regard, we aimed to infer possible significant effects of the season (four classes: spring, summer, autumn and winter), the lactation status (two classes: dry and lactating), the bedding temperature, the temperatures and the relative humidities at the heights of 0.10 and 1.30 m above the bedding material, the ambient temperature, the moisture content, the days between the complete renewal of bedding and the sample date, the bedding area and the CBP group (4 groups) on phenotypic observations for the total amount of TAS (log10 cfu/g) and on concentrations of single TAS species. The statistical model was shown as following:

y_ij_ = µ + eff_i_ + e_ij_, (2)

where y_ij_ is the phenotypic observations for log10 (cfu/g) or TAS concentration; μ is the overall mean; eff_i_ is one of the aforementioned fixed effects in separate runs; e_ij_ is the random residual effects. The fixed effects were tested separately, because only 13 samples were available in the analyses. For the TAS concentration, we calculated a ratio of the number of specific TAS species to the total number of TAS. For example, in one sample, *Bacillus licheniformis* was detected twice, and *Laceyella sacchari* was identified once. Hence, the concentration of *Bacillus licheniformis* for this sample was 0.67 (2/3). In this study, the significance level was defined as *p* < 0.05.

## 3. Results and Discussion

### 3.1. CBP Bedding Material Parameters

In general, the observed bedding temperatures (17.93 to 56.57 °C) in combination with moisture content agreed with those summarised by Leso et al. [2].

The means for the observed bedding temperatures, temperatures and relative humidities at the heights of 0.10 and 1.30 m above the bedding, outside temperatures, moisture contents and TAS concentrations are listed in Table 1.

The mean pack temperature Tbed over all CBP bedding material samples was 39.93 °C, ranging from 17.93 °C in winter 2019 to 56.57 °C in autumn 2018. In winter 2019, we analysed only the group of dry cows (because of the comprehensive and time-consuming analyses in the molecular laboratory). In this regard, we focused on the group of dry cows, because in this group at this sampling date, limited disturbing farm management practices were observed. The quite small animal number in this group explained the low bedding temperature. The desired high bedding temperatures of 55 to 65 °C, promoting pathogen destruction [11,18] and composting efficiency [11,15,18], were rarely achieved. Black et al. [11] referred to this phenomenon as a “semi-composting” process. However, the low pack temperatures observed are in line with results from the studies reviewed by Leso et al. [2]. They may also be due to the mechanical processing of the bedding material in the process of bedding aeration as described before [14] and the fans in the barn. For this reason, some Dutch producers modified the pack management during colder months, reducing both cultivating frequency and depth [14]. Leso et al. [2] indicated that the low and partly wide range of temperatures is not sufficient to support a full composting process. Furthermore, the effect that non-TAS species are prevented from growing by high temperatures [2,5] is probably lost. However, Leso et al. [20] recommended an optimal ventilation management, which allows a high drying rate of the bedding even at lower bedding temperatures when biological activities of the microbial populations and heat production are reduced. Furthermore, Leso et al. [2] mentioned that greater differences between the pack and ambient temperatures indicate that the pack is biologically active, which was also observed for the samples in the present study.

In autumn 2018, an optimal composting process was achieved for the first time. Here, bedding temperatures were high, while temperatures at the heights of 0.10 and 1.30 m above the bedding material and ambient temperatures were low.

Because moisture content and bedding temperature are negatively correlated, humid conditions in the bedding lead to decreasing temperatures and thus a loss of aerobic conditions. The anaerobic conditions are unfavourable for TAS. The measured moisture contents over all CBP samples were on average 59.55% (48.23% to 68.8%), reflecting the recommended range of 40%−60% [3,17,19]. Moisture contents between 30% and 35% also inhibit microbial activity, ceasing the composting process [18,19]. Composting requires sufficient moisture for active microbial activity, but extremely high values hinder aeration [3]. The observed moisture contents in the present study are comparable with those reported by Black et al. (56.10% ± 12.40%) [11] and lower in comparison to those identified by Eckelkamp et al. (59.90% to 76.60%) [5]. The longer the bedding material has been used and the more the animal manure has been supplemented, the higher the moisture content, visible in spring 2018 with the highest moisture contents. The decrease in moisture content in the summer of 2018 might be due to the significant increase in outdoor temperature, leading to an increased drying rate. Additionally, the water-holding capacity of the air increased with higher ambient temperatures, reflected by the measured reduced relative humidities at the heights of 0.10 and 1.30 m above bedding material (Table 1), causing more moisture to evaporate from the CBP [11,47]. The bedding material was completely replaced in autumn 2018 (i.e., 44 days before the autumn sampling), explaining the lower moisture contents during this period and the ongoing increase until winter 2019. The compositions of the bedding differed between the measurements in winter 2017 and autumn 2018, which affected water absorption capacities and bedding temperatures while outdoor conditions were quite constant. In winter 2017, the bedding consisted of wood chips and spelt husks. In winter 2018, the bedding consisted of wood chips, spelt husks, shredded brushwoods and broken roots. Furthermore, the heat developed by the composting process in autumn 2018 can increase the drying rate as already described by Leso et al. [2], ideally supported by optimised stable ventilation [20]. The increase in drying rate implies a decreased moisture content. This in turn can extend the bedding usability [5].

### 3.2. Bacterial Growth/Identification

**Figure 1:**
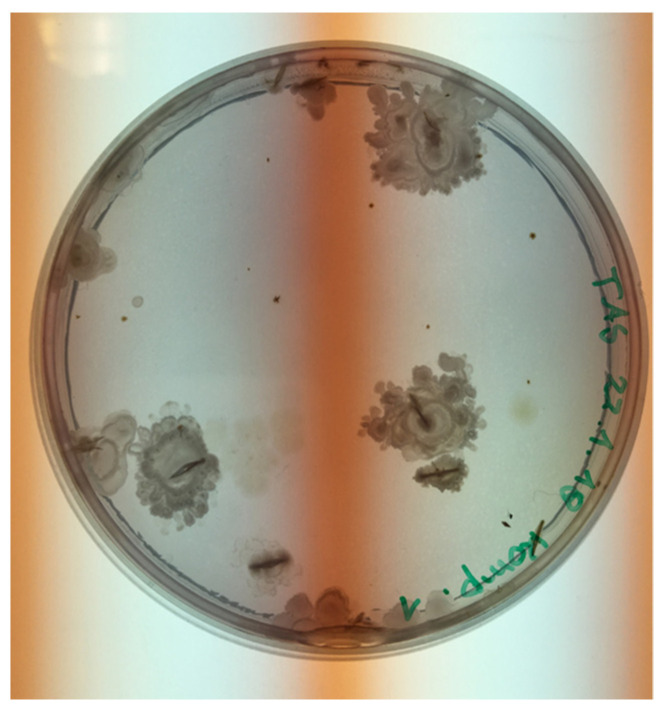
Grown thermophilic aerobic sporeformers (TAS) colonies on a Dextrose Tryptone Agar plate after incubation at 55 °C for 48 h.

Figure 1 shows the grown TAS colonies on the DTA medium. No TAS colonies were observed on the uninoculated agar plates.

Counting the colonies resulted in a mean TAS amount of 4.11 log10 cfu/g across all 13 samples.

The highest log10 cfu/g values were identified in the first winter period 2017 directly after the first cows moved into the CBP with a 5.74 log10 cfu/g bedding material in the lactating group and a 5.86 log10 cfu/g bedding material in the group of dry cows (Table 1). The TAS amount in the unused bedding material from the store in winter 2017 was 1.96 log10 cfu/g, being lower than log10 cfu/g-values of the unused wood chips (<4.00–6.70 log10 cfu/g) identified by Driehuis et al. [26]. Nevertheless, we also identified TAS spores in the stored bedding, as already shown [26,28]. Driehuis et al. [26] postulated that TAS in stored products are due to the self-heating of the bedding material in the storage area. This may explain the highest number of TAS amounts in winter 2017. The bedding material used later was usually bedded in immediately after delivery.

Lowest cfu numbers were identified in spring and autumn 2018 with 3.48 and 3.23 log10 cfu/g bedding materials (Table 1), respectively. In spring 2018, this is likely due to the significantly increased moisture content of the bedding material larger than 60%, which was above the optimal range of 40%–60% [3,17,19]. These values are generally lower than the range from 6.50 to 8.90 log10 cfu/g as indicated by Leso et al. [2] but comparable to reports by Driehuis et al. [26]. In autumn 2018 (sample 12), the TAS concentration may be low for two reasons: firstly, the days between the complete renewal of bedding and the sample date was only 44 days, and secondly, the CPB4 group consisted of a smaller number of animals. This implies the decreasing inclusion of excreta into the bedding material, which delayed the composting process and thus the growth of TAS.

For adjacent colony PCR, we picked well-differentiated individual, not overlapping colonies. Due to the previous heat treatment, we could assume that only TAS colonies grew on the DTA medium. Since we were not able to identify which TAS species grew on the basis of the colonies, we tried to pick all colonies that looked different in order to be able to identify as many TAS species as possible. This is partly the reason for the rather heterogeneous number of the picked colonies/sample (Table 1). Additional measurements of the DNA concentrations using Nanodrop 1000 spectrophotometers supported the selection of the colonies to be sequenced.

### 3.3. PCR and Sequence Analyses

**Figure 2:**
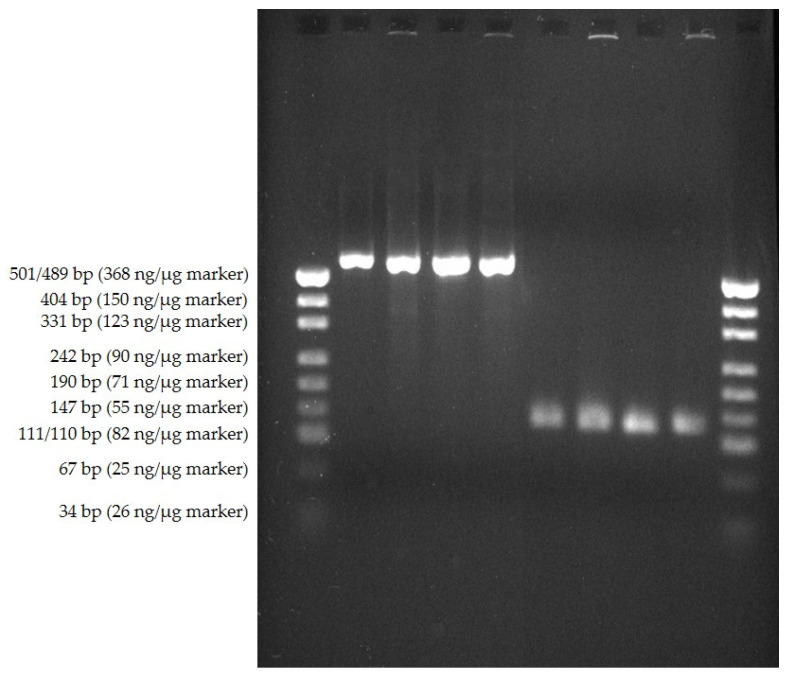
Agarose gel electrophoresis of colony PCR: slots 1 and 10: marker; slots 2–5: colony PCR products of PCR-no. 1 (Table 2) of different picked colonies, amplifying V1, V2 and V3 of the 16S rRNA-gene; slots 6–9: colony PCR products of PCR-no. 2 (Table 2) of different picked colonies, amplifying V6 of the 16S rRNA-gene.

The colony PCR products of the two PCR (Table 2) for the adjacent sequencing of parts of the 16S rRNA-gene are presented in Figure 2.

**Figure 3:**
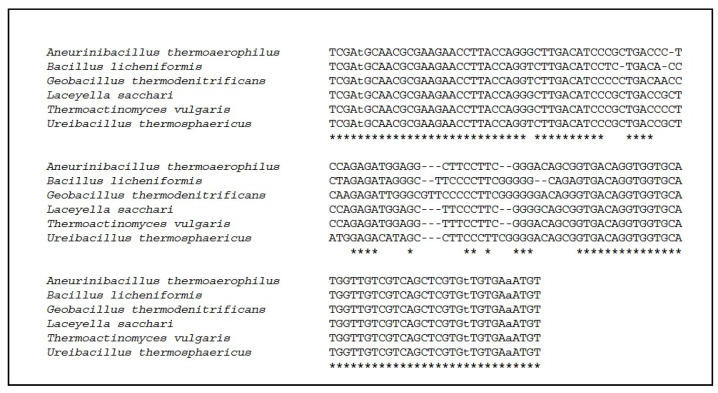
Alignment of the sequences of PCR-no. 2 (Table 2) of the hypervariable region V6. Stars mark matching sequences across all 6 TAS species. Small letters in the primer regions at the beginning and the end of the sequences mark sequence differences due to fixed universal primers (Table 2).

The sequencing of the two parts of the 16S rRNA-gene of the 99 colonies identified the TAS species *Aneurinibacillus thermoaerophilus*, *Bacillus licheniformis*, *Geobacillus thermodenitrificans*, *Laceyella sacchari*, *Thermoactinomyces vulgaris* and *Ureibacillus thermosphaericus*. The resulting sequences of the hypervariable regions V1 to V3 (PCR-no. 1, Table 2) were submitted to GenBank under Acc. No. OK090768 to OK090773 for the respective TAS species sequence of the hypervariable region V6 (PCR-no. 2; Table 2), as presented in Figure 3, displaying markable sequence differences between the six identified TAS species.

Accordingly, Charbonneau et al. [25] represented the four genera *Geobacillus*, *Bacillus*, *Ureibacillus* and *Aneurinibacillus* by 16S rRNA-gene sequencing. In 61.22% of all colonies, *Bacillus licheniformis* was identified. Apart from sample 8, *Bacillus licheniformis* displayed the highest proportion of TAS in all bedding samples at all sampling dates in all CBP groups (Table 3). *Thermoactinomyces vulgaris* and *Laceyella sacchari* were the second and third most common TAS species with 16.33% and 10.20%, respectively. The wide distribution of *Bacillus licheniformis* has already been described, e.g., also in fodders, leading to its occurrence in faeces [29,30]. The roughage component was the same in all CBP groups, which may explain the wide distribution of *Bacillus licheniformis.* Hence, it is a facultative thermophile bacterium growing in a wide temperature range [21]. The bedding temperature showed a wide range from 17.93 to 56.57 °C, with a mean of 39.93 °C. Therefore, this adaptive TAS species is favoured.

*Bacillus licheniformis*, *Geobacillus thermodenitrificans* and *Ureibacillus thermosphaericus* have previously been found in CBP samples [25,26]. *Laceyella sacchari* and *Thermoactinomyces vulgaris* are frequently isolated from outdoor environments such as soil and grassy pastures and thus show a wide distribution in cattle environments [28]. Consequently, the broad species spectrum is expected. Furthermore, *Aneurinibacillus thermoaerophilus* is already described in animal manure compost [25] and is amongst *Bacillus licheniformis*, *Ureibacillus thermophaericus* and *Geobacillus* spp. possibly *thermodenitrificans* present in cow milk samples [30].

When comparing the TAS species occurrences of different CBP groups across adjacent seasons between which no exchange of the bedding material took place, it is noticeable that some TAS species could not be found again (Table 1 and Table 3). One reason for this may be that the living conditions developed to the disadvantage of one or the other TAS species in the course of the progressive usage of the bedding material, so that the bacteria could no longer spread in the following season or even disappeared. In a direct comparison of the sampling seasons winter 2017 and spring 2018, the moisture content of the bedding material and the relative humidities at the heights of 0.10 and 1.30 m above the bedding material substantially changed, which may be decisive in this respect. In addition, due to the continuous crushing of the bedding material by the cows, smaller materials slipped to the bottom of the CBP [1]. Consequently, it is possible that TAS that adhered to these smaller bedding material components were no longer detected. The sequencing of parts of the 16S rRNA-gene identified the three most abundant TAS from the CBP *Bacillus licheniformis* (45.46%), *Laceyella sacchari* (18.18%) and *Thermoactinomyces vulgaris* (36.36%) in cubicles. Hence, these TAS species occur ubiquitously on the farm. Accordingly, Driehuis et al. [26] stated that the type of housing (CBP or cubicle housing) is of minor importance for the occurrence of TAS.

*Bacillus licheniformis* and the *Geobacillus* species, for example, are classified as non-pathogenic [48], and also Black et al. [49] described that *Bacillus* bacteria are rarely the cause of mastitis. Moreover, the desired high bedding temperatures in CBP promote pathogen destruction and a reduction of mastitis-specific bacteria [5,11,18]. Therefore, there is no increased risk of mastitis for cows kept in CBP compared to those kept in cubicles. However, TAS which form resistant biofilms are able to survive from milk pasteurisation and result in the loss of milk product quality [36,37,49]. Therefore, they are of particular importance.

### 3.4. Effects on the TAS Amount and Concentration

Effects of external influences such as bedding temperature or moisture content and ambient temperature on the amount of TAS or on the occurrence of specific TAS species in CBP bedding samples have, to our knowledge, not yet been carried out. Black et al. [49] investigated the levels of *Coliforms*, *Escherichia coli*, *Streptococci*, *Staphylococci* and *Bacillus* spp. For this purpose, the bedding material samples were incubated at 35 °C for different times. TAS were not cultivated with them. However, they identified associations between bacterial concentrations and space per cow, moisture content and temperature of the bedding material [49]. Therefore, we expected that these parameters also have effects on the amount and concentration of TAS.

#### 3.4.1. Effects on the Amount of TAS (log10 cfu/g Bedding Material)

**Figure 4:**
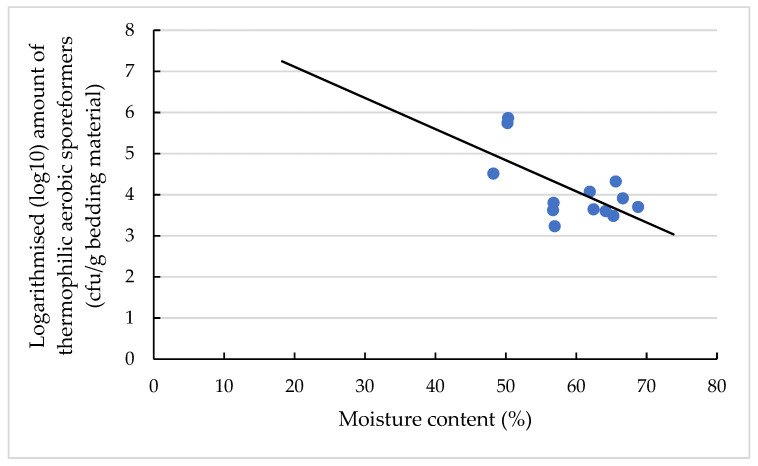
Levels of logarithmised (log10) amount (cfu/g bedding material) of TAS in dependency of moisture content. The individual dots reflect the individual samples, and the black line is the regression line calculated with the equation y = −0.08x + 8.62 and a coefficient of determination of 0.34.

**Figure 5:**
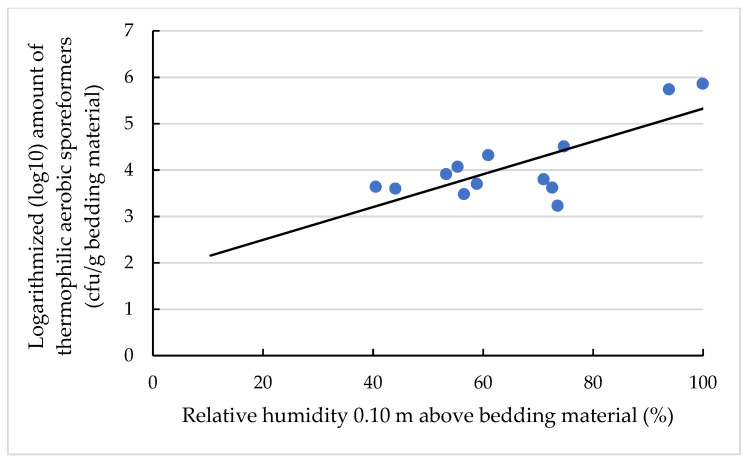
Levels of logarithmised (log10) amount (cfu/g bedding material) of TAS in dependency of relative humidity at the height of 0.10 m above the bedding material. The individual dots reflect the individual samples, and the black line is the regression line calculated with the equation y = −0.04x + 1.79 and a coefficient of determination of 0.57.

The moisture content of the bedding material (*p* < 0.05; Figure 4), relative humidities at the heights of 0.10 m (*p* < 0.05; Figure 5) and 1.30 m (*p* < 0.05) above the bedding material and the season of sampling (*p* < 0.01) significantly affected the amount of TAS in the bedding material of the CBP.

The amount of TAS in winter (5.31 ± 0.31 log10 cfu/g) was significantly higher than in spring (3.79 ± 0.27 log10 cfu/g), summer (3.62 ± 0.38 log10 cfu/g) and autumn (3.79 ± 0.27 log10 cfu; Table S1). The significant effect of the sampling season (p < 0.01) is presumably due to the seasonal interaction between the ambient temperature and the relative humidity, which in turn also have indirect effects on bedding temperature and moisture content. In agreement with those reported by Eckelkamp et al. [5] and Black et al. [11], we found the increasing bedding temperature and the decreasing moisture content with increasing stable and ambient temperature. As a result, rising ambient temperatures led to drier bedding areas, implying clean cows with reduced risk for mastitis infections [11].

A significant association between the TAS amount and the moisture content (Figure 4) is due to the fact that the oxygen required for the aerobic processes of these bacteria is reduced under wet conditions [2,3], resulting in a reduced TAS amount when the moisture content was above 60%. The negative relationship between the moisture content and the TAS amount is indicated by the regression coefficient of −0.08 ± 0.03 (Table S2).

In addition, the relative humidities at the heights of 0.1 m (Figure 5) and 1.3 m above the bedding material showed significant effects on the TAS amount. This is due to a direct effect on the drying rate, which in turn influences the moisture content of the bedding material [2].

However, we did not find significant effects of the bedding temperature, the temperatures at the heights of 0.10 and 1.30 m above the bedding material, the ambient temperature, animal group, lactation status, days between the complete renewal of bedding and the sample date and the bedded area per cow on cfu/g values (partly shown in Table S1).

The bedded area per cow is one of the most important parameters in a CBP design [2]. At all times, cows in all groups had more than the minimum recommended 7.40 m^2^/cow of an open bedded packing area available. If more space is provided per animal, the average amount of urine and faecal water per m^2^ is lower, implying a decrease in moisture to be evaporated per m^2^ [11]. Nevertheless, we could not detect any significant association between the stocking density and the TAS amount. The positive health and well-being effects of CBP for lactating cows are also evident in dry cows [50]. In principle, the handling of the bedding area is easier for dry cows than for lactating cows, as the amount of moisture per animal is much lower than for high-yielding cows [51]. However, there was no significant impact of lactation status on the amount of TAS (Table S1).

The fact that the period between the complete renewal of the litter and the sampling day showed tendencies (*p* < 0.1; regression coefficient of −0.01 ± 0.00; Table S2) on the TAS concentration may be related to the fact that the fresh litter partly already contains TAS spores [26,28]. In the present study, we identified 1.96 log10 cfu TAS per g bedding material in one sample from the stock. In addition, the bedding moisture increased with increasing usage time of the bedding material as described above, so that the moisture content then had an indirect effect again.

#### 3.4.2. Effects on the TAS Concentrations of the Different TAS Species

The characteristic TAS concentration provides information about which the bacterium grew most strongly under which bedding material and temperature conditions, due to the fact that the corresponding TAS could then be identified most frequently via 16S rRNA-gene sequencing. For example, in sample 8, *Thermoactinomyces vulgaris* formed the majority of the 3.64 log10 cfu/g bedding material, whereas Bacillus licheniformis, the most prevalent bacterium overall, showed a lower concentration (Table 3). In addition, *Geobacillus thermodenitrificans* was identified in 4 of the total 16 picked colonies in sample 8. This corresponded to a concentration of 25.00%, which was higher than the total *Geobacillus thermodenitrificans* concentration of 6.12% over all samples. Accordingly, we can conclude that the conditions prevailing in sample 8 with a 62.46% moisture content, at a 40.17 °C bedding temperature and a very high ambient temperature are favourable for the two TAS species *Thermoactinomyces vulgaris* and *Geobacillus thermodenitrificans*. In sample 2, on the other hand, the conditions prevailed the occurrence of almost all TAS species. Here, five from the in total six TAS species could be identified.

Although season had a significant effect on the total amount of TAS, we did not detect a significant effect on the TAS concentration of a single species. Nevertheless, there are tendencies that, for example, *Ureibacillus thermosphaericus* only occurs in the winter and autumn seasons with cold ambient temperatures. However, the differences in TAS species distribution between sampling seasons were not significant (*p* > 0.05). Similar results are shown for *Laceyella saccheri*, where the days between the complete renewal of the bedding material and the sample date showed a negative tendency on the presence of this TAS (*p* < 0.1). With regard to the lactation status, ls means for the TAS concentrations were very similar, and the lactation status effect was not significant (Table S1). Nevertheless, *Thermoactinomyces vulgaris* was more frequent in the groups of lactating cows than in the group of dry cows. Concentrate feeding adapted to the lactation status of the cows could possibly play a role in this regard. The dry cows on the farm received no concentrates, thus increasing contents of crude fibre in their ration. As already described [28], grain can be a source of *Thermoactinomyces vulgaris*. Spores located in fodders are unaffected during the passage through the gastrointestinal tract of the cow and are excreted in faeces [29,30], probably causing the contamination of the CBP bedding material and explaining also the presence of *Thermoactinomyces vulgaris* in the group of first-lactation and late-lactating cows kept in cubicles.

**Figure 6:**
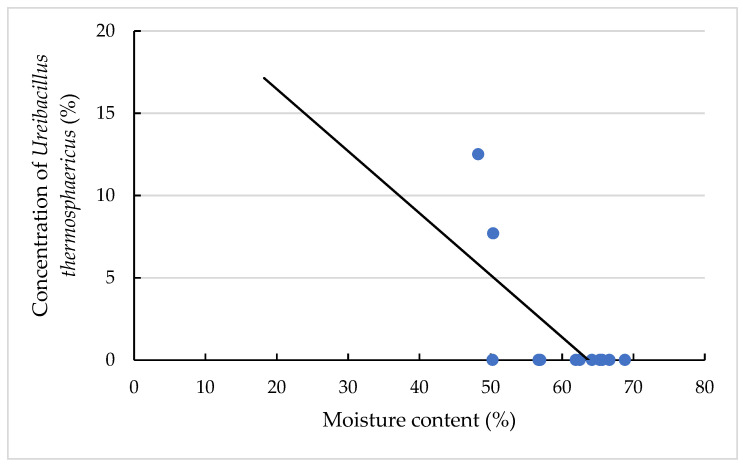
Concentration of *Ureibacillus thermophaericus* (%) in dependency of moisture content. The individual dots reflect the individual samples, and the black line is the regression line calculated with the equation written as: y = −0.38x + 24.01 and a coefficient of determination of 0.38.

In the group of dry cows, over all samples, all six TAS species could be identified. The wide TAS species spectrum in sample 2 can be explained by the fact that at 2 of the 3 different sampling sites Tbed was clearly above 40 °C (the mean value of the Tbed was lowered by a very low value at one transit point). This could favour the growth of further obligate thermophilic bacteria such as *Aneurinibacillus thermoaerophilus* and *Geobacillus thermodenitrificans* especially in comparison to sample 1, which is similar in terms of Tbed (total) and moisture content (Table 1). The results in Table S2 showed a significant effect of the moisture content of the bedding material on the presence of *Ureibacillus thermophaericus* (*p* < 0.05), which is additionally presented in Figure 6. In two bedding samples with moisture contents of 48.23% and 50.33%, *Ureibacillus thermophaericus* was identified. The regression coefficient of the scaled moisture content on concentration of *Ureibacillus thermophaericus* was −0.38 ± 0.13 (Table S2).

**Figure 7:**
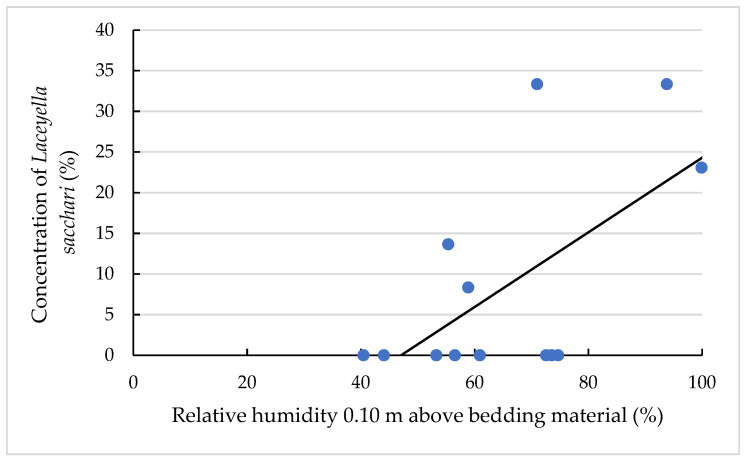
Concentration of *Laceyella sacchari* (%) in dependency of moisture content. The individual dots reflect the individual samples, and the black line is the regression line calculated with the equation written as: y = 0.46x − 21.61 and a coefficient of determination of 0.38.

Furthermore, the relative humidities at the heights of 0.10 m (Figure 7) and 1.30 m above the bedding material had a significant (*p* < 0.05) effect on *Laceyella sacchari* concentration, explaining its absence in summer season, for example, when the relative humidity seemed to be too low for the growth of this TAS species.

The tendencies for bedding temperature and bedding area per cow were shown for the occurrence of *Aneurinibacillus thermoaerophilus* (*p* < 0.1). Interestingly, the obligate thermophilic bacterium *Aneurinibacillus thermoaerophilus* was found mainly in samples with a rather low bedding temperature (17.93 to 34.77 °C). This is also shown by the negative regression coefficient of −0.49 ± 0.22 (Table S2).

Overall, we showed that the recorded bedding material characteristics such as moisture content and temperature as well as the barn management characteristics such as relative humidity significantly affected the amount of TAS and the TAS species concentrations. Further studies in this context are imperative to define management recommendations for setting optimal TAS contents by considering the specific farm characteristics. Beffa et al. [16] already mentioned the challenges for reducing *Bacillus* spp. while maintaining active composting. Bacteria are always present in CBP, which is desirable for effective composting. In order to reduce the risk of TAS transfer from the teats into milk, it is therefore particularly important to keep the moisture content of the bedding material at an optimum level.

In this context, it should be noted that the CBP bedding composition parameters are also beneficial for fungal growth [52], whereas previous studies showed a decrease in the amount of fungi during thermophilic composting processes [53]. However, microbial and molecular genetic studies on fungal growth in CBP bedding materials should also be conducted in subsequent studies including a possible influence of the fungal growth on the TAS growth and vice versa.

## 4. Conclusions

We identified different TAS species in CBP groups with different concentrations. The sequencing of parts of the 16S rRNA-gene led to the identification of six different TAS species, with *Bacillus licheniformis* being the most common species. Especially, the moisture content of the bedding material and the relative humidity above the bedding material have significant influences on the TAS concentration and, to some extent, on the TAS species concentration. Therefore, an optimal compost management is a prerequisite for a functional dairy farming in CBP with a dry lying surface and an optimal composting process with a moderate microbial, especially the TAS amount and the TAS concentration. Since three of the six identified TAS species were also found in cubicles, it can be assumed that these TAS species occur ubiquitously on the farm. Further research is needed to: a) examine the effects of these ubiquitous TAS on udder health; b) study their effects on milk product quality; and c) give clear CBP management recommendations.

In the present study, only samples from one German CBP farm were considered. The selected large-scale CBP herd is a model herd for compost bedding in Germany, and we considered repeated measurements from all seasons. Nevertheless, for detailed validations and TAS-farm characteristic association analyses, it is imperative to consider a larger number of CBP herds and to focus on a longitudinal data structure in further herds.

## Figures and Tables

**Table 1:** Moisture content, means for temperatures and relative humidities (standard deviation in brackets) and thermophilic aerobic sporeformers (TAS) concentrations in 13 different compost-bedded pack barns (CBP) samples.

Sample Number	Sampling Season	CBP Group	Days ^a^	Mc ^b^ (%)	Tbed ^c^ (°C)	Thigh ^d^ (°C)	RHhigh ^d^ (%)	Tdown ^e^ (°C)	RHdown ^e^ (%)	Tamb ^f^ (°C)	Bed ^g^ (m^2^)	Picked Colonies (n)	TAS (log10)
1	winter 2017	CBP1-lact. ^h^	8	50.24	31.13 (±13.80)	7.92 (±0.54)	93.78 (±18.37)	8.16 (±0.86)	98.70 (±2.94)	7.40	8.90	3	5.74
2		CBP4-dry	8	50.33.	34.77 (±16.09)	8.00 (±0.17)	99.90 (±0.00)	8.20 (±0.10)	99.90 (±0.00)		13.18	13	5.86
3	spring 2018	CBP1-lact.	113	68.80	36.08 (±8.46)	12.10 (±0.81)	51.50 (±2.76)	12.22 (±0.66)	58.87 (±4.72)	14.30	8.42	12	3.70
4		CBP2-lact.	108	66.63	33.96 (±7.36)	12.99 (±0.78)	49.26 (±3.97)	13.01 (±0.92)	53.28 (±4.60)		7.80	7	3.91
5		CBP3-lact.	80	65.28	34.96 (±7.18)	12.08 (±0.27)	52.38 (±2.40)	12.13 (±0.27)	56.53 (±4.26)		8.31	1	3.48
6		CBP4-dry	113	61.93	32.83 (±16.80)	11.60 (±0.20)	52.37 (±1.23)	11.70 (±0.20)	55.37 (±1.36)		12.99	22	4.07
7	summer 2018	CBP1-lact.	248	64.19	42.12 (±6.84)	31.94 (±0.32)	40.87 (±1.21)	31.88 (±0.42)	44.06 (±0.42)	34.20	8.82	2	3.60
8		CBP2-lact.	248	62.46	40.17 (±7.85)	32.52 (±0.35)	38.79 (±1.80)	32.64 (±0.20)	40.48 (±3.56)		8.01	16	3.64
9	autumn 2018	CBP1-lact.	44	48.23	55.97 (±17.27)	6.66 (±0.42)	70.16 (±2.42)	6.72 (±0.43)	74.67 (±3.38)	6.20	8.66	8	4.51
10		CBP2-lact.	44	56.71	46.31 (±14.15)	6.52 (±2.25)	68.27 (±2.16)	7.29 (±0.63)	72.57 (±3.26)		9.44	4	3.62
11		CBP3-lact.	44	56.79	56.33 (±5.99)	6.12 (±0.43)	68.03 (±1.23)	6.24 (±0.57)	70.99 (±1.91)		9.14	6	3.80
12		CBP4-dry	44	56.94	56.57 (±0.64)	5.83 (±0.51)	72.10 (±3.11)	6.07 (±0.76)	73.53 (±4.97)		11.11	1	3.23
13	winter 2019	CBP4-dry	121	65.62	17.93 (± =3.36)	5.63 (±0.06)	60.73 (±0.97)	5.67 (±0.06)	60.93 (±0.49)	7.90	21.96	4	4.32

^a^ Days between the complete renewal of bedding and the sample date. ^b^ Moisture content. ^c^ Mean temperatures of the bedding material at the 9 (lactating cows) and 3 (dry cows) sampling points. ^d^ Mean temperatures and relative humidities at a height of 1.30 m above the bedding at the 9 (lactating cows) and 3 (dry cows) sampling points, respectively. ^e^ Mean temperatures and relative humidities at a height of 0.10 m above the bedding at the 9 (lactating cows) and 3 (dry cows) sampling points, respectively. ^f^ Ambient temperature. ^g^ Bedded area/cow. ^h^ lact. = lactating.

**Table 2:** Polymerase chain reaction (PCR) conditions of colony PCR for the adjacent sequencing of parts of the 16S rRNA-gene.

PCR-No.	Primers	Sequences (Position in GenBank Acc. No. X60623 ^a^)	Amplified Hypervariable Region	Reference
1	27F	5′-GAGTTTGATCCTGGCTCA-3′ (3–20)	V1, V2 and V3	[27,39]
	V3R	5′-CGTATTACCGCGGCTGCTG-3′ (539–521)		
2	V6F	5′-TCGAtGCAACGCGAAGAA-3′ (963–980)	V6	[39]
	V6R	5′-ACATtTCACaACACGAGCTGACGA-3′ (1084–1061)		

^a^ In comparison to GenBank Acc. No. X60623, primers V6F and V6R showed some small sequence differences signed by small letters.

**Table 3:** Frequencies (%) of the identified TAS species after the cultivation of 13 different compost-bedded pack barn (CBP) bedding samples and 16S rRNA-gene sequencing.

		TAS Species					
Sample Number	Sampling Season	*A. thermoaeroph.* ^a^	*B. lichenif.* ^b^	*G. thermodenit.* ^c^	*L. sacch.* ^d^	*T. vulg.* ^e^	*U. thermosph.* ^f^
1	winter 2017	-	0.67	-	0.33	-	-
2		0.08	0.46	0.15	0.23	-	0.08
3	spring 2018	-	0.84	-	0.08	0.08	-
4		0.29	0.71	-	-	-	-
5		-	1.00	-	-	-	-
6		-	0.68	-	0.14	0.18	-
7	summer 2018	-	1.00	-	-	-	-
8		-	0.31	0.25	-	0.43	-
9	autumn 2018	-	0.50	-	-	0.37	0.13
10		-	0.75	-	-	0.25	-
11		-	0.67	-	0.33	-	-
12		-	1.00	-	-	-	-
13	winter 2019	0.25	0.75	-	-	-	-

^a^ Aneurinibacillus thermoaerophilus. ^b^ Bacillus licheniformis. ^c^ Geobacillus thermodenitrificans. ^d^ Laceyella sacchari. ^e^ Thermoactinomyces vulgaris. ^f^ Ureibacillus thermosphaericus.

## Data Availability

The sequence data of the hypervariable regions V1 to V3 of the identified TAS species are openly available at https://www.ncbi.nlm.nih.gov/nuccore under Acc. No. OK090768 to OK090773.

## Supplementary Materials

The following are available online at https://www.mdpi.com/article/10.3390/ani11102890/s1, Table S1: Least-square means of amount of TAS (log10 cfu/g bedding material) and TAS concentration by season, lactation status and CBP group (standard errors in brackets), Table S2: Regression coefficients of days between the complete renewal of bedding and the sample date (days), moisture content (Mc), bedding temperature (Tbed), temperatures at the heights of 1.30 m (Thigh) and 0.10 m (Tdown) above the bedding material, relative humidities at the heights of 1.30 m (RHhigh) and 0.10 m (RHdown) above the bedding material, ambient temperature (Tamb) and bedded area/cow on the amount and the concentration of TAS (standard errors in brackets).
