# Supplementary material for: Identification of Thermophilic Aerobic Sporeformers in Bedding Material of Compost-Bedded Dairy Cows Using Microbial and Molecular Methods
Source: Animals (Basel). 2021 Oct 4;11(10):2890. doi: 10.3390/ani11102890 (PMC8532821; doi:10.3390/ani11102890)
Supplement: Supplementary file 1 [file animals-11-02890-s001.zip › animals-1366846-supplementary.pdf]

Table S1. Least-square means of amount of thermophilic aerobic sporeformers (TAS; log10 cfu/g bedding material) and TAS concentration by season, lactation status and compost-bedded pack barn (CBP) group (standard errors in brackets).

Trait	Season				Lactation status		CBP group			
	Spring	Summer	Autumn	Winter	Dry	Lactating	1	2	3	4
Amount of TAS	3.79[a] (0.26)	3.62[a] (0.37)	3.79[a] (0.26)	5.31[b] (0.31)	4.37 (0.42)	4.00 (0.28)	4.39 (0.43)	3.72 (0.50)	3.64 (0.61)	4.37 (0.43)
Aneurinibacillus thermoaerophilus	7.14 (5.11)	0.00 (7.23)	0.00 (5.11)	10.90 (5.90)	8.17 (5.10)	3.17 (3.40)	0.00 (5.17)	9.52 (5.97)	0.00 (7.31)	8.17 (5.17)
Bacillus licheniformis	80.74 (11.46)	65.63 (16.21)	72.92 (11.46)	62.61 (13.23)	72.33 (11.06)	71.59 (7.37)	75.00 (11.30)	59.23 (13.05)	83.33 (15.99)	72.33 (11.30)
Geobacillus thermodenitrificans	0.00 (3.61)	12.50 (5.11)	0.00 (3.61)	5.13 (4.17)	3.85 (4.08)	2.78 (2.72)	0.00 (4.06)	8.33 (4.69)	0.00 (5.75)	3.85 (4.06)
Laceyella sacchari	5.49 (6.56)	0.00 (9.28)	8.33 (6.56)	18.80 (7.58)	9.18 (6.82)	8.33 (4.55)	10.42 (6.84)	0.00 (7.90)	16.67 (9.67)	9.18 (6.84)
Thermoactinomyces vulgaris	6.63 (7.88)	21.88 (11.15)	15.63 (7.88)	0.00 (9.10)	4.55 (7.98)	12.73 (5.32)	11.46 (7.75)	22.92 (8.95)	0.00 (10.96)	4.55 (7.75)
Ureibacillus thermosphaericus	0.00 (2.09)	0.00 (2.95)	3.13 (2.09)	2.56 (2.41)	1.92 (2.04)	1.39 (1.36)	3.13 (2.12)	0.00 (2.45)	0.00 (3.00)	1.92 (2.12)

[a, b] Different letters represent significant differences ($P < 0.05$).

Table S2. Regression coefficients of days between complete renewal of bedding and sample date (Days), moisture content (Mc), bedding temperature (Tbed), temperature 1.30 m (Thigh) and 0.10 m (Tdown) above bedding material, relative humidity 1.30 m (RHhigh) and 0.10 m (RHdown) above bedding material, ambient temperature (Tamb), and bedded area/cow on amount of thermophilic aerobic sporeformers (TAS) and TAS concentrations (standard errors in brackets).

Trait	Days	Mc	Tbed	Thigh	RHhigh	Tdown	RHdown	Tamb	Bedded area/cow
Amount of TAS	-0.01 (0.00)	-0.08* (0.03)	-0.03 (0.02)	-0.03 (0.03)	0.03* (0.01)	-0.03 (0.03)	0.04* (0.01)	-0.03 (0.02)	0.05 (0.06)
Aneurinibacillus thermoaerophilus	0.01 (0.04)	0.50 (0.42)	-0.49 (0.22)	-0.18 (0.32)	-0.04 (0.16)	-0.19 (0.33)	-0.06 (0.17)	-0.13 (0.30)	1.28 (0.68)
Bacillus licheniformis	0.00 (0.08)	1.33 (0.84)	0.04 (0.56)	-0.20 (0.69)	-0.25 (0.34)	-0.21 (0.69)	-0.24 (0.36)	-0.08 (0.64)	-0.02 (1.65)
Geobacillus thermodenitrificans	0.03 (0.03)	-0.12 (0.34)	-0.05 (0.21)	0.43 (0.22)	-0.01 (0.13)	0.44 (0.22)	-0.03 (0.13)	0.37 (0.21)	-0.12 (0.61)
Laceyella sacchari	-0.08 (0.04)	-0.90 (0.51)	0.00 (0.35)	-0.45 (0.40)	0.44* (0.17)	-0.46 (0.41)	0.46* (0.18)	-0.47 (0.37)	-0.12 (1.02)
Thermoactinomyces vulgaris	0.06 (0.06)	-0.44 (0.68)	0.39 (0.40)	0.50 (0.49)	-0.23 (0.25)	0.52 (0.50)	-0.23 (0.26)	0.42 (0.46)	-1.00 (1.19)
Ureibacillus thermosphaericus	-0.02 (0.01)	-0.38* (0.13)	0.10 (0.10)	-0.10 (0.12)	0.09 (0.06)	-0.10 (0.13)	0.10 (0.06)	-0.11 (0.11)	-0.02 (0.31)

* Significant regression coefficient ($P < 0.05$).
